# Cognitive function and brain activation before and after transcutaneous cervical vagus nerve stimulation in healthy adults: A concurrent tcVNS-fMRI study

**DOI:** 10.3389/fpsyg.2022.1003411

**Published:** 2022-11-11

**Authors:** Han Zhang, Zhiwei Guo, Yun Qu, Yu Zhao, Yuxuan Yang, Juan Du, Chunlan Yang

**Affiliations:** ^1^Department of Rehabilitation Medicine, West China Hospital of Sichuan University, Chengdu, China; ^2^Department of Rehabilitation Medicine, Nanchong Central Hospital, The Second Clinical Hospital of North Sichuan Medical College, Nanchong, China; ^3^School of Rehabilitation, West China Medical College, Sichuan University, Chengdu, China; ^4^Sichuan Provincial Key Laboratory of Rehabilitation Medicine, Sichuan University, Chengdu, China; ^5^Brain Function Rehabilitation and Development Institute, Nanchong Central Hospital, The Second Clinical Hospital of North Sichuan Medical College, Nanchong, China

**Keywords:** transcutaneous cervical vagal nerve stimulation, cognition, memory, functional magnetic resonance imaging, neural activity

## Abstract

Transcutaneous vagus nerve stimulation, which involves the application of electrical currents to the cervical (tcVNS) or auricular (taVNS) branches of the vagus nerve, may be a potential treatment for improving cognitive dysfunction. taVNS may improve cognitive performance in healthy adults, and fewer studies have been performed on the effects of tcVNS on cognition in healthy subjects. We conducted a randomized, single-blind, crossover-controlled trial to investigate the effects of tcVNS stimulation on cognitive function and neural activity in the brains of healthy adults. This study provides support for further tcVNS studies for the treatment of cognitive impairment. Twenty-one participants were randomly divided into two groups, A and B. Group A received tcVNS first and then sham-tcVNS, while group B received the intervention in the reverse order, receiving sham stimulation first and then true stimulation. All subjects were required to perform cognitive function tests before and after receiving intervention, and functional magnetic resonance imaging (fMRI) was performed concurrently during the intervention. We hypothesized that tcVNS would have an effect on the cognitive performance of the subjects and alter the neural activity of the brain. The present study showed that tcVNS had beneficial effects on cognitive performance, mainly improving memory and language skills and attention. tcVNS intervention produced significant spontaneous neural activity in the calcarine gyrus, fusiform gyrus, lingual gyrus, and parahippocampal gyrus of the brain. Future tcVNS/fMRI trials will need to explore the effects of changes in stimulus parameters on the neural activity response of the brain.

## Introduction

The vagus nerve (VN) is the longest and most widely distributed brain nerve. It can regulate the function of organs through a complex neuroendocrine immune network and is involved in the regulation of inflammation, mood and pain in humans (Gallowitsch-Puerta and Pavlov, 2007; Wang et al., 2021). Traditionally, vagus nerve stimulation (VNS) is always used to treat patients with refractory seizures or depression by surgically implanted electrodes (Ben-Menachem, 2001; Lei and Duan, 2021), but its invasive nature limits its use and research. Compared to invasive VN stimulation, noninvasive VN stimulation, namely, transcutaneous VNS (tVNS), is used in research by transcutaneous stimulation of the cervical (tcVNS) or auricular (taVNS) with fewer adverse effects (Colzato and Beste, 2020).

In recent years, studies have reported that tVNS can be used as a potential treatment for improving cognitive function (Cai et al., 2019; Hakon et al., 2020; Thakkar et al., 2020). However, the potential mechanism of tVNS on cognitive performance in populations, including healthy individuals, remains unclear. Most studies of tVNS have involved stimulating the auricular branch of the VN. It has been suggested that taVNS may improve cognition in healthy adults, and these studies have focused on cognitive processes, involving in sequential learning and response selection, defined as sequences of actions ordered to achieve a task goal (Jongkees et al., 2018), post error slowing, which refers to people tend to slow down after committing a mistake (Sellaro et al., 2015), Divergent thinking, also known as creative thinking, is the ability to generate new ideas for open-ended problems (Colzato et al., 2018), Speed and flexibility in switching between tasks (Borges et al., 2020), executive functions and memory functions (Klaming et al., 2022). Functional magnetic resonance imaging (fMRI) also shows that taVNS changes the blood flow of somatosensory areas, the nucleus tractus solitarius (NTS), limbic lobe, amygdala, hippocampus, insula, precentral gyrus, thalamus, hypothalamus, anterior cingulate cortex (ACC), and the left dorsolateral prefrontal cortex (DLPFC), suggesting that taVNS causes activation in emotionally and cognitively relevant brain regions (Badran et al., 2018).

Little is known about the effects of tcVNS on cognition in healthy subjects. In a study where tcVNS was applied to patients under sleep deprivation stress (McIntire et al., 2021), it showed significantly better performance in arousal and multitasking than sham stimulation. It was also found that tcVNS increased activation of the ACC and hippocampus in patients with posttraumatic stress disorder (Wittbrodt et al., 2021). Therefore, it is largely unknown whether tcVNS will have behavioral effects similar to those of taVNS by activating brain activity in healthy adults.

The short-wisconsin card sorting test (WCST) and verbal fluency test (VFT) are tools and can be used to assess cognitive function in patients with neurological disorders such as dementia, traumatic brain injury, and stroke (Maseda et al., 2014; Delgado-Alvarez et al., 2020; Miles et al., 2021). We performed a tcVNS/fMRI study to determine the afferent pathways of the VN in healthy adults receiving left cervical stimulation. Therefore, in this study, WCST and VFT were used to assess the cognitive abilities of the subjects, and fMRI was used to examine the underlying neural mechanisms of tcVNS on cognitive function. The results revealed that tcVNS significantly improved overall cognitive, memory and language functions. In addition, altered activation was found in the precuneus lingual gyrus, left parahipoparietal gyrus, and left hippocampal gyrus in healthy subjects.

## Materials and methods

**Figure 1:**
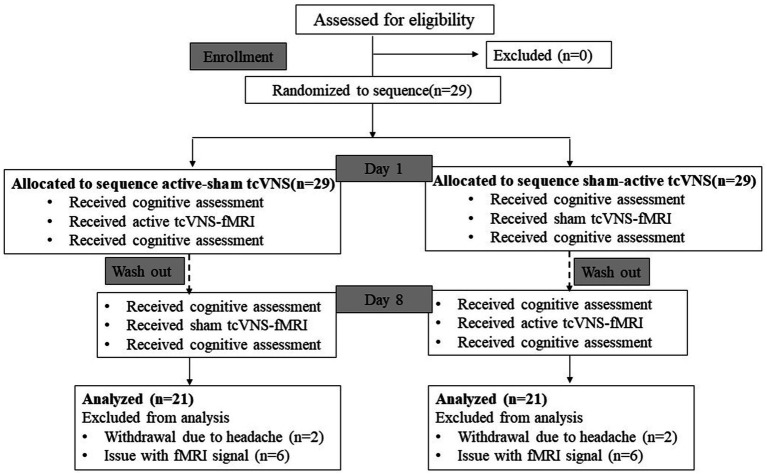
Diagram of the study. tcVNS, transcutaneous cervical vagal nerve stimulation; fMRI, functional magnetic resonance imaging.

We conducted a randomized, single-blind, crossover-controlled trial to investigate the effects of transcutaneous cervical VN stimulation (tcVNS) on cognitive function and brain activation in healthy adults. Participants were randomly divided into two groups, A and B. Group A received tcVNS first and then sham-tcVNS, while group B received the intervention in the reverse order, receiving sham stimulation first and then true stimulation. The two interventions were delivered at least 7 days apart to avoid carry over effects. All subjects were required to perform cognitive function tests before and after receiving intervention, and fMRI scans were performed concurrently during the intervention (Figure 1). Scans were obtained at a 3 T MRI scan system dedicated to the Center for Functional Brain Imaging Research at the institution. The study was approved by the institutional ethics committee. All participants gave written informed consent prior to the experiment.

### Participants and inclusion criteria

We performed a power analysis for multi-factorial repeated-measures designs with G*power3.1. We obtained an effect size of 0.43 based on a previous study with similar study design. We then conducted a power analysis with an expected effect size of 0.43, an alpha error of 0.05, a power of 0.86, with a sample size of 20. A total of 21 healthy students (57.1% males and 42.9% females; age range 20–23 years; mean ± SD: 21.38 ± 0.962 years) with a mean duration of education of 15.3 years from a local university were enrolled in this study. All these subjects were right-handed. Participants were excluded if they had a history of heart disease, substance abuse or dependence, psychiatric medication, history of neurological or psychiatric disorders, pregnancy, and the presence of an active implanted device (e.g., pacemaker, cochlear implant). They were asked not to smoke, exercise, consume food, drink alcohol, or consume caffeine for at least 2 h prior to participation.

### fMRI scanning

The MRI scans were all performed in a Philips 3.0 T system with a 32-channel head coil. Subjects were placed supine in the MRI scanner, and a foam pad was used to stabilize their head to minimize movement. Subjects underwent resting-state fMRI(rs-fMRI) twice, during which the tcVNS or sham tcVNS was provided concurrently with the second fMRI scan. All fMRI data were acquired by using an echo-planar imaging (EPI) sequence with the following parameters: TR = 2000 ms, TE = 30 ms, field of view = 240.0 mm × 240.0 mm, matrix = 64 × 64, flip angle = 90°, thickness/interspace = 3.75 mm/0.0 mm, slices = 33, and voxel size = 3.75 mm × 3.75 mm× 3.75 mm, with interleaved slice scan order. Each scan obtained 255 volumes continuously. During the rs-fMRI scanning, subjects were asked to stay awake, relax with their eyes closed and remain motionless as much as possible. All patients also underwent a high-resolution 3D T1-weighted anatomical scan: TR = shortest, TE = shortest, field of view = 240.0 mm × 240.0 mm, flip angle = 20°, matrix = 256 × 256, voxel sizes = 1 mm × 1 mm × 1 mm, for a total of 170 slices.

### tcVNS parameters and stimulation

**Figure 2:**
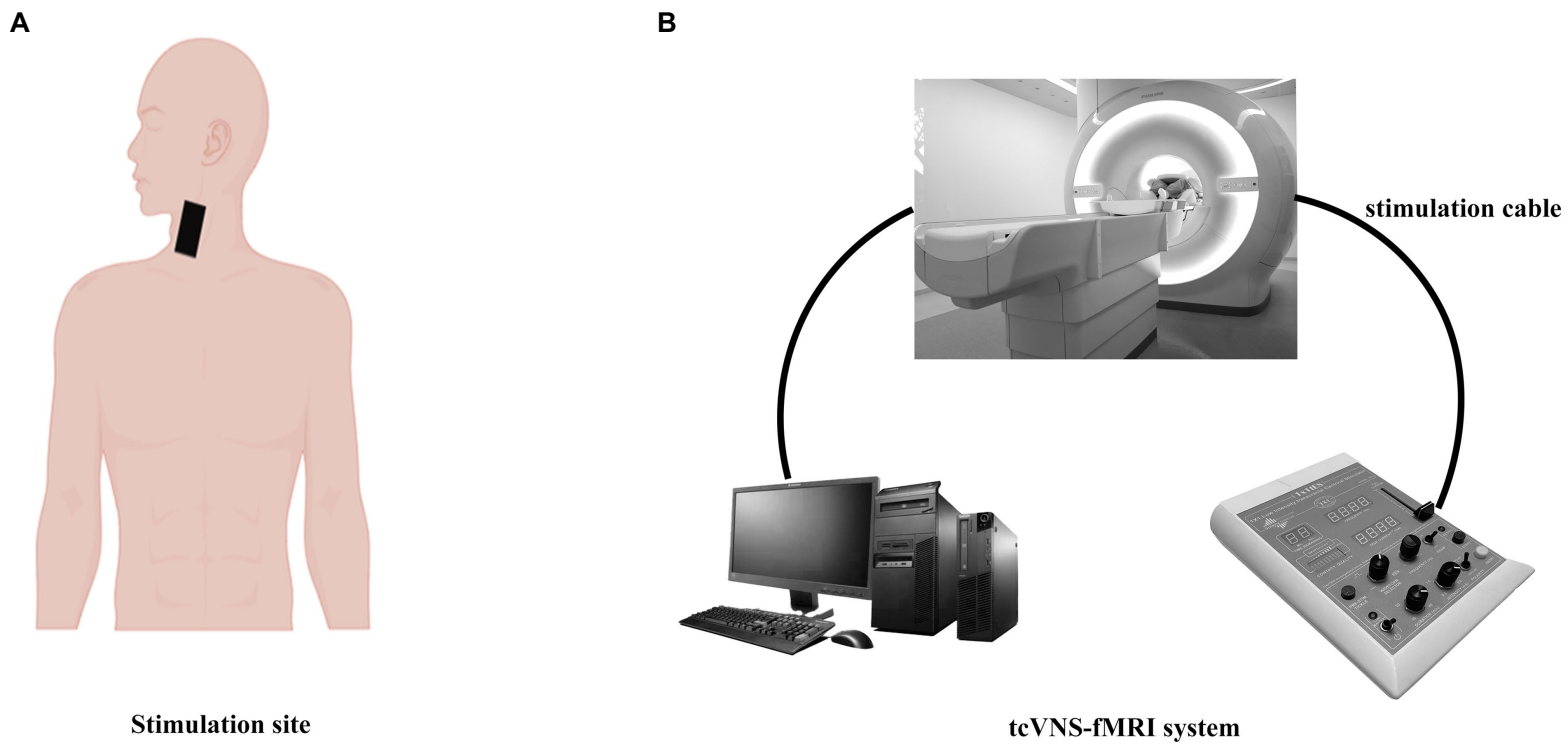
Transcutaneous cervical vagus nerve stimulation (tcVNS) with concurrent fMRI.

The stimulator (Soterix, 1 × 1tES) was placed on the left cervical VN, which lasted for 8 min and was repeated at a frequency of 25 Hz (24 V for tcVNS, 4.5 V for sham), 2 mA, 0.5 ms pulse width. Previous studies have shown that stimulation of distal cervical locations using low voltage does not result in vagal excitation (Lerman et al., 2019). A 20 V nVNS placed directly on the carotid artery has been shown to activate the VN (Frangos and Komisaruk, 2017; Mourdoukoutas et al., 2018; Klaming et al., 2022). Both the tcVNS and sham stimulation protocols consisted of an initial rise period of 30 s, followed by a steady peak stimulation. Then, steady peak VNS stimulation was provided to each subject during the second fMRI examination (Figure 2).

### Evaluation

#### The short Wisconsin card sorting test

The short version of the Wisconsin card sorting test (WCST) was generated from a modification of the WCST and consists of 4 stimulus cards and 60 response cards (Miles et al., 2021). For the subject to take the test, the participant must select a matching card from the four stimulus cards based on a criterion (the matching criterion can be a color, a figure, or a number) that matches the response card. For example, a response card with two blue triangles could be matched based on color (blue), form (triangles), or number (two). After each reaction, the subject receives feedback (i.e., “correct” or “incorrect”), which can be used to establish the correct sorting rule. After subjects produce 10 correct matches in a row, the sorting criteria change without any warning, which requires a flexible cognitive shift. There is no time limit on the test, which ends when the individual has sorted all cards or has reached six correct sorting criteria. Once completed, the sum and percentage of the following scores are calculated: total number of action (TA), total number of correct response (CR), number of correct classifications (CC), number of responses required to complete the first classification (TCFC), total number of errors (TE), number of persistent errors (PE), number of nonpersistent errors (NPE), and total time (TIME). It is one of the most widely known tools for assessing executive function in a variety of populations.

#### Verbal fluency test

The VFT is a neuropsychological test that can be used to assess memory and language function (Hung et al., 2016). Subjects are asked to say as many words as possible in 1 min based on semantic, phonological, and action categories. The number of correct, number of errors, number of series, number of subcategory conversions, and number of correct per 15 s in 60 s will be recorded in each category.

#### Processing of the fMRI images

For each subject, two runs of rs-fMRI images were obtained in each session, of which one was obtained during tcVNS or sham stimulation. The preprocessing of all of the data was performed by using SPM 12^1^ software package, by which the slice timing and spatial head motion realignment, normalization, and smoothing were conducted. Prior to the preprocessing procedure, the first 5 volumes of the fMRI datasets of each patient were discarded to eliminate the magnetization equilibrium effects and allow the participants to adapt the circumstances.

After preprocessing, the time signal of each voxel of the images was scaled by dividing each time point’s value by the mean value of the whole brain image to remove the linear trend. Then, a bandpass filter (0.01–0.08 Hz) was used to reduce the interference of the drift and noise on the scaled signal. After these time domain preprocessing steps, the amplitude of low-frequency fluctuation (ALFF) and mean ALFF were calculated for each voxel. All these processing steps were conducted by using the REST 1.8 software package.^2^

### Data processing and analysis

Differences in demographic and behavioral characteristics between the groups were assessed using two-sample *t* tests. Paired sample *t* tests were used to assess differences in performance on executive function tasks before and after receiving the intervention in each group. A general linear model with mixed effects was conducted to examine the effects of tcVNS and sham tcVNS on the TA, CR, CC, TCFC, TE, PE, NPE, and TIME in WCST and also number of correct, number of errors, number of series, number of subcategory conversions, and number of correct per 15 s in 60s in VFT as the dependent variable, intervention program and phase of implementation as fixed factors, and subject number as a random factor. We applied the Bonferroni multiple comparison correction (Holm, 1979) in order to control for false discovery rate (FDR). It is necessary to determine whether the sphericity assumption is violated before testing for the interaction of within factors. if *p* < 0.05, the sphericity assumption is not satisfied and we use the Greenhouse–Geisser method for correction. In each group, a 2-tailed paired *t* test was employed to detect significant changes in ALFF after tcVNS stimulation to investigate the changes in spontaneous brain activity induced by tcVNS stimulation. Between groups, a two-sample *t* test was used to explore the difference between tcVNS and sham stimulation on the brain activities and the AlphaSim was used for correction. Then, according to the ALFF results of the tcVNS group, Pearson correlation analysis was employed to calculate the correlation coefficient between altered ALF*F* values after tcVNS stimulation and altered cognitive scores. *p* < 0.05 was considered to be statistically significant.

## Results

### Subject characteristics

All subjects completed this trial. No significant differences were detected between the tcVNS and sham-tcVNS groups in terms of age or sex.

### Cognitive function performance

Compared to the sham-tcVNS, subjects in the tcVNS had a significant main effect on the number of errors in the action categories task (−0.24 ± 0.54 vs. 0.14 ± 0.48, *F* = 5.43, *p* = 0.025) and the number of correct errors from the 45^th^ second to 60^th^ second in the phonological category task (PC-correct45) (2.05 ± 2.6 vs. 0.29 ± 2.55, *F* = 4.272, *p* = 0.049) in the VFT. A significant main effect of period on the TIME (−24 ± 28.32 vs. −15.67 ± 29.89, *F* = 4.54, p = 0.04) of the WCST test, total number of correct responses (1.29 ± 4.595 vs. 2.48 ± 5.68, *F* = 6.31, *p* = 0.016), number of correct responses from 45 s to 60s (SC-correct45) (1.14 ± 4.69 vs. 2.71 ± 4.57, *F* = 5.084, *p* = 0.03) and number of series (2.71 ± 4.92 vs. 1.71 ± 5.82, *F* = 4.35, *p* = 0.044) in the semantic category, total number of correct responses in phonological category task (3.57 ± 3.84 vs. 2.14 ± 3.38, *F* = 5.09, p = 0.03), and number of correct responses in the first 15 s of the action category task (0.33 ± 1.43 vs. 0.57 ± 2.0, *F* = 4.6, *p* = 0.039) in the VFT was found, showing that the performance improved over time. There was a significant interaction effect between intervention and stage on the TA (*F* = 4.19, *p* = 0.048), TIME (*F* = 4.74, *p* = 0.036; Table 1). There were no significant random effects.

**Table 1:** Cognitive tasks: results of the general linear model (GLM) related to cognitive performance.

	*F* value	*p* Value		*F* value	*p* Value
TA			PC-correct		
Stage	0.002	0.963	Stage	5.092	0.03^♀^
Intervention	1.286	0.264	Intervention	1.023	0.318
Stage*intervention	4.187	0.048^♀^	Stage*intervention	1.302	0.261
TIME			PC_ correct45		
Stage	4.54	0.04^♀^	Stage	1.404	0.243
Intervention	0.481	0.492	Intervention	4.272	0.048^♀^
Stage*intervention	4.74	0.036^♀^	Stage*intervention	0.014	0.905
SC-correct			AC-errors		
Stage	6.313	0.016^♀^	Stage	0.803	0.376
Intervention	1.304	0.261	Intervention	5.429	0.025^♀^
Stage*intervention	0.603	0.442	Stage*intervention	3.212	0.081
SC-correct45			AC-correct15		
Stage	5.084	0.03^♀^	Stage	4.595	0.039^♀^
Intervention	2.15	0.151	Intervention	0.009	0.926
Stage*intervention	1.171	0.286	Stage*intervention	2.224	0.144
**SC-series**					
Stage	4.345	0.044^♀^			
Intervention	0.099	0.755			
Stage*intervention	0.000	0.993			

TA, total number of actions; SC-correct, total number of correct answers in 60 s in semantic category; SC-correct45, number of correct responses from 45 s to 60 s in semantic category; SC-series, number of series in semantic category; PC-correct, total number of correct answers in 60 s in phonological category; AC-errors, total number of errors in 60 s in action category; AC-correct15, number of correct responses in the first 15 s in the action category; PC_correct45, number of correct responses from 45 s to 60 s in phonological category.

^♀^*p* < 0.05.

### Spontaneous activity changes induced by tcVNS

**Figure 3:**
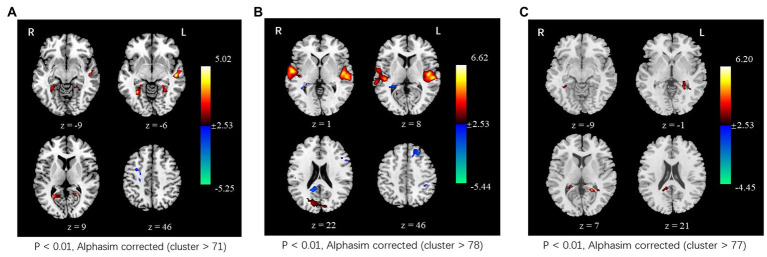
Changes in spontaneous neural activity induced by tcVNS. **(A)** Comparison results of ALFF between concurrent tcVNS stimulation and rest in the tcVNS group (*p* < 0.01, AlphaSim corrected, cluster >71). **(B)** Comparison results of ALFF between concurrent sham stimulation and rest in the sham group (*p* < 0.01, AlphaSim corrected, cluster >78). **(C)** Comparison results of ALFF between the tcVNS and sham groups (*p* < 0.01, AlphaSim corrected, cluster >77).

In the tcVNS group, compared to the resting state, the ALFF value significantly increased in the bilateral calcarine gyrus, lingual gyrus, fusiform gyrus, parahippocampal gyrus, and left middle temporal gyrus and significantly decreased in the right frontal gyrus during tcVNS stimulation. In the sham group, significantly increased ALFF was also observed in the bilateral superior temporal gyrus, bilateral middle temporal gyrus, bilateral cuneus gyrus, and left precuneus; significantly decreased ALFF was detected in the left superior frontal gyrus, left middle frontal gyrus, left inferior parietal lobe, and right precuneus. In addition, relative to the sham group, higher ALFF was found in the left precuneus lingual gyrus, left parahippppcampal gyrus, and left hippocampus gyrus (Figure 3).

### Correlation between neural activity and cognitive task performance

The Pearson analysis revealed significant correlation between neural activity and cognitive task performance. In the WCST task performance, it showed that there was a significant correlation between the altered ALFF of the right calcarine gyrus and TCFC performance changes (*r* = 0.47, *p* = 0.042), between the altered ALFF of the right fusiform gyrus and the performance changes of PE (*r* = −0.493, *p* = 0.032), between the altered ALFF of the left lingual gyrus and the changes of TA (*r* = 0.476, *p* = 0.040), PE (*r* = −0.470, p = 0.042), between the altered ALFF of the left calcarine gyrus and the performance changes of PE (*r* = −0.572, *p* = 0.011), and between the altered ALFF of the right frontal gyrus and the changes of TC (*r* = −0.473, *p* = 0.041), CC (*r* = −0.552, *p* = 0.014).

**Figure 4:**
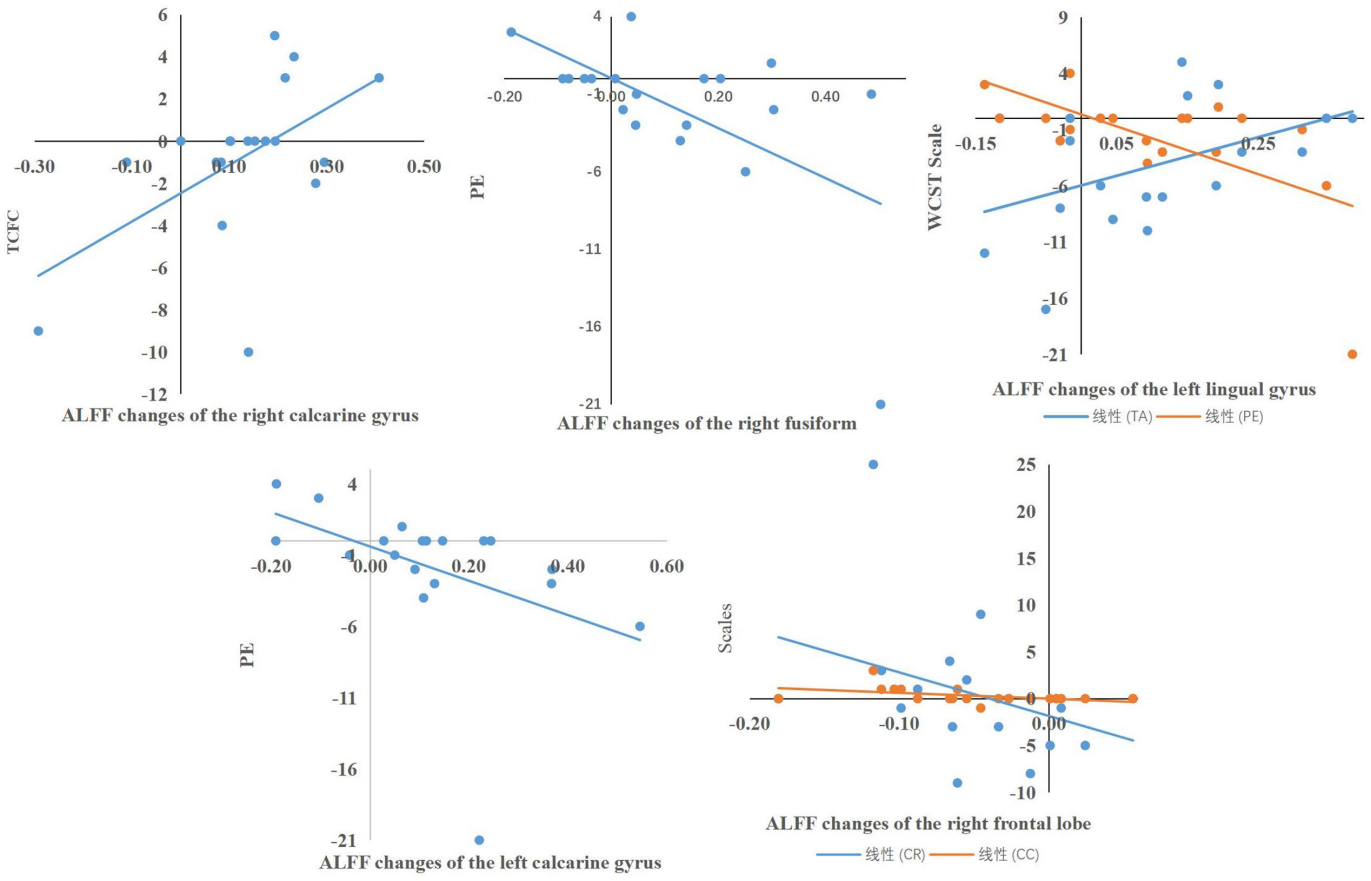
Correlation between the variability of ALFF and the variability of WCST task performance.

**Figure 5:**
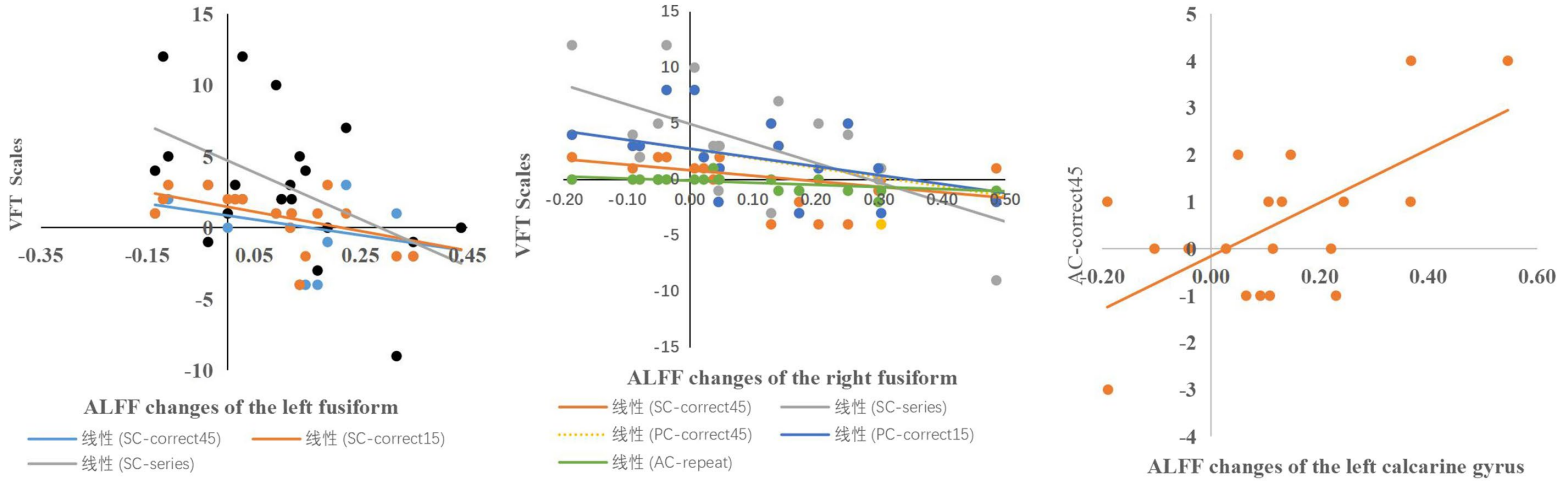
Correlation between the variability of ALFF and the variability of VFT task performance.

In the VFT task performance, a significant correlation between the altered ALFF of the right fusiform gyrus and the performance of SC-correct45 (*r* = −0.535, *p* = 0.018), SC-series (*r* = −0.569, *p* = −0.026), PC-correct45 (*r* = −0.509, *p* = 0.026), the number of corrects in the first 15 s of the phonological category task (*r* = −0.495, *p* = 0.031), the number of repeat in action category task (*r* = −0.608, *p* = 0.006) was found. There was significant correlation between the altered ALFF of the left fusiform gyrus and the performance of SC-correct45 (*r* = −0.492, *p* = 0.033), number of correct responses in the first 15 s in the semantic category(*r* = −0.600, *p* = −0.007), SC-series (*r* = −0.468, *p* = 0.043), between the altered ALFF of the left calcarine gyrus and performance of the 45th second to 60th second in action category (*r* = 0.467, *p* = 0.044) (Figures 4, 5).

## Discussion

We used the tcVNS-fMRI paradigm to observe the effects of tcVNS on cognitive performance and brain activation in 21 healthy individuals. Our results found that tcVNS improved overall cognitive, memory and language performance in healthy subjects.

The present study showed that tcVNS had a beneficial effect on cognitive performance. After receiving tcVNS, subjects were able to recall more words in the phonological category task, made fewer errors in the semantic category task, and showed higher accuracy when performing the VFT test compared to the sham tcVNS. VFT reflects a variety of cognitive components, including correct number, clustering, and transformation. The evaluation of the different cognitive components can be applied to analyse the cognitive demands underlying each task. VFT performance is influenced by cognitive processes such as attention-executive function, episodic memory, and language (Maseda et al., 2014; Hung et al., 2016; Whiteside et al., 2016; Kim et al., 2021). Whiteside et al. found that VFT is primarily a language-based task, particularly in relation to both phonological and semantic fluency. However, de Paula et al. indicated that VFT was more strongly associated with executive functions of working memory, inhibitory control, and cognitive flexibility, primarily involving the two basic cognitive processes of clustering and transformation in verbal fluency performance. Among category tasks, semantic fluency is explained more by the memory function, while phonological fluency is more related to the executive function. Based on these behavioral observations, we found that tcVNS improved memory and language performance. This is consistent with the results of a previous study that showed that noninvasive cervical VNS improves performance on visuospatial problem solving and recognition tasks by increasing alertness and attention (Klaming et al., 2022). The intervention phase should not interfere with cognitive performance because of the randomized intervention order (tcVNS was randomly delivered as the first and second intervention), but our results nevertheless show that the intervention phase has a main effect on subjects’ cognitive performance, which can be explained by a practice effect. All subjects were to be tested twice on the same cognitive task at each stage, and subjects could perform better cognitively on the second stage of the test due to repeated practice.

Our results showed that tcVNS created significant spontaneous neural activity throughout the calcarine gyrus, fusiform, lingual gyrus, and parahippocampal regions compared to sham stimulation among healthy young adults. ALFF is a common indicator of spontaneous neuronal activity in resting-state fMRI studies (Xiao and Dong, 2021; Wang et al., 2021). Among them, fractional low-frequency amplitude can directly reflect the amplitude of hemorrhagic oxygen level-dependent signal change, which in turn shows the intensity of spontaneous activity of brain neurons (Liang et al., 2021). In the study, the brain regions with elevated low-frequency amplitudes in the tcVNS compared to the sham-tcVNS included the left precuneus, lingual gyrus, left parahippcampal gyrus, and left hippocampal gyrus. Recent functional imaging results in healthy subjects suggest that the precuneus plays a central role in a wide range of highly integrated tasks, including visuospatial imagery, contextual memory, information processing with self-centred mental imagery strategies, and metacognition, which may be associated with consciousness (Cavanna and Trimble, 2006; Soldevila-Matías et al., 2022). An investigative study of stroke by Kumral et al. found that infarcts occurring in the anterior precuneus may result in impaired consciousness (25%), impaired self-processing (42%), and visuospatial impairment (58%), while lesions in the posterior precuneus caused deficits in episodic and semantic memory (33%). Injuries to the entire precuneus then involved disorders of consciousness (Kumral et al., 2021). The parahippocampal gyrus serves as the main cortical input to the hippocampus and is closely associated with emotion and cognition (Christopher et al., 2015). The lingual gyrus is a brain structure associated with visual processing that plays a role in analysing logical conditions and encoding visual memories (Suzuki et al., 2022). The results of our study are inconsistent with those of Klaming et al. (2022), who found that tcVNS improves cognitive-behavioral performance in healthy adults but does not cause significant changes in blood oxygen level-dependent (BOLD) signals. The reason for the inconsistency may involve differences in study design. We controlled for the characteristics of the subjects’ education level, age, and sex ratio to reduce the effects of these characteristics on cognitive performance and brain activation patterns. Additionally, we synchronized fMRI testing during the course of subjects receiving 8 min of tcVNS, whereas another study synchronized fMRI testing during the course of receiving a cognitive task after tcVNS stimulation, potentially detecting brain activation for the cognitive task rather than tcVNS.

Additionally, we analysed the correlation between the performance of cognitive tasks and neural activity in the brain. A correlation was found between cognitive performance and ALFF values in the right calcarine gyrus, the right fusiform gyrus, the left lingual gyrus, the left fusiform gyrus, the left calcarine gyrus, and the right frontal gyrus. In this study, significant changes were found in both the fusiform region and the performance of AC_series and PC_correct45 in the VFT after tcVNS intervention. Previous studies have also found that the fusiform is associated with cognitive changes (Gardini et al., 2009; Lebedeva et al., 2015; Li et al., 2021), suggesting that the area of the brain may be a potential mechanism of cognitive enhancement in tcVNS.

Our study still has some limitations. First, the sample size was small. In addition, although we have balanced the effects between intervention order and subjects, practice effects resulting from neuropsychological test properties may have confounded the findings, and these findings underscore the difficulty of designing a reliable neuropsychological study that also considers the effects of recurrent test tasks on subjects’ cognitive performance in future studies. Due to methodological limitations, our findings did not reveal any brainstem activation in regions that are known to be the entry points of the VN into the central nervous system (CNS), such as the NTS or Locus Coeruleus (LC). The human LC is a very small brain region to be imaged using advanced imaging techniques (Badran et al., 2018). Our scan sequence was optimized to capture whole-brain hemodynamics at a voxel size of 3.75 mm × 3.75 mm × 3.75 mm, limiting the ability to capture NTS and LC structures. Future studies should employ specific advanced tcVNS-fMRI midbrain and brainstem imaging as well as whole-brain scanning.

In addition, future studies may consider setting up no stimulation as a control group to ensure that sham stimulation has no effect on the results. Finally, because stimulation parameters vary from study to study, it is unclear which parameter is ideal for achieving the best outcome of cognitive improvement. Therefore, studies are also needed to optimize stimulus parameters such as intensity (repetition of stimuli, duration of stimuli) and stimulus timing settings (including stimulation before or after the cognitive task, or performing simultaneous stimulation of the cognitive task); to design different recognition tasks for different dimensions of cognitive function, e.g., the beneficial effects of tcVNS may be limited to different stages of memory function such as encoding, storage, recall recognition, or different types of information (i.e., verbal vs. nonverbal, affective vs. neutral, implicit vs. explicit).

## Conclusion

These findings suggest that tcVNS is associated with better performance on memory and language function than sham stimuli and produces significant neural activity effects on the calcarine gyrus, fusiform gyrus, lingual gyrus, and parahippocampal gyrus. These findings are similar to those of previous tcVNS imaging studies, but there are differences in their effects on neural activity in the brain. Future tcVNS/fMRI trials will need to explore the effects of changes in stimulus parameters on the neural activity response of the brain.

## Data availability statement

The original contributions presented in the study are included in the article/supplementary material, further inquiries can be directed to the corresponding author.

## Ethics statement

The studies involving human participants were reviewed and approved by the Ethics Committee of Nanchong Central Hospital. The patients/participants provided their written informed consent to participate in this study.

## Author contributions

HZ and Zw-G prepared the manuscript and contributed to the study design. YZ conducted the cognitive assessment. Yx-Y, JD, and Cl-Y performed the fMRI scanning. YQ reviewed and approved the manuscript for final submission. All authors contributed to the writing of the article and approved the submitted version.

## Funding

This study was supported by the National Key R&D Plan (No. 2017YFC1308504) and Youth Innovation Research Project of Sichuan Provincial Medical (No. Q20036).

## Conflict of interest

The authors declare that the research was conducted in the absence of any commercial or financial relationships that could be construed as a potential conflict of interest.

## Publisher’s note

All claims expressed in this article are solely those of the authors and do not necessarily represent those of their affiliated organizations, or those of the publisher, the editors and the reviewers. Any product that may be evaluated in this article, or claim that may be made by its manufacturer, is not guaranteed or endorsed by the publisher.

## Footnotes

^1^ http://www.fil.ion.ucl.ac.uk/spm

^2^ http://www.restfmri.net
